# Durability of Students’ Learning Strategies Use and Beliefs Following a Classroom Intervention

**DOI:** 10.3390/bs15050706

**Published:** 2025-05-21

**Authors:** Ezgi M. Yüksel, C. Shawn Green, Haley A. Vlach

**Affiliations:** 1 Department of Psychology, University of Wisconsin-Madison, Madison, WI 53706, USA; cshawn.green@wisc.edu; 2 Department of Educational Psychology, University of Wisconsin-Madison, Madison, WI 53706, USA; hvlach@wisc.edu

**Keywords:** learning strategies, effective learning strategies, instruction, direct experience, durability of intervention

## Abstract

When students choose their own learning strategies, they often rely on ineffective methods, such as rereading and cramming, which have limited long-term benefits. To improve learning outcomes, previous interventions have utilized explicit instruction about effective strategies and direct experience with those strategies, though with mixed success. Yüksel et al. demonstrated that combining both approaches could foster initial improvements in students’ understanding and use of effective learning strategies. In Study 1, we examined the long-term effects of this combined intervention by contacting participants six months later to assess the stability of outcomes. In Study 2, we extended the scope by surveying all students who had enrolled in the intervention section over the past five years. Participants were asked about their use and perceived effectiveness of various strategies. In both studies, quantitative measures were complemented with open-ended questions to gain deeper insights into study behaviors and obstacles to adopting effective strategies. While students retained an understanding of the effectiveness of various strategies and reported using ineffective strategies less frequently, the adoption of more effective strategies did not show a significant increase. However, compared to the business-as-usual group, the intervention group did not experience a decline in their use of effective strategies. These results suggest that while explicit instruction and experience can enhance knowledge, long-term behavior change remains difficult. Reported obstacles—such as time constraints, limited resources, procrastination, and prioritizing short-term gains—align with metacognitive theories of desirable difficulties and help explain why students still favor less effortful strategies, despite knowing more effective ones that require greater effort and delayed rewards.

## 1. Introduction

College students exhibit wide-ranging differences in their ability to effectively learn and retain new material (Cassidy, 2012; Chen et al., 2000). While inherent cognitive abilities account for some of the divergence in learning outcomes (e.g., Lyons & Zelazo, 2011; Ahmed et al., 2019), research has indicated that disparities in learning performance are also related to variations in how students approach learning (Bowen & Wingo, 2012; Bartoszewski & Gurung, 2015; Hassanbeigi et al., 2011; Hartwig & Dunlosky, 2012). Unfortunately, students typically receive little or no formal instruction on the effectiveness of various learning practices. As such, they are largely left to their own devices with respect to how to approach learning. Research shows that students frequently utilize a host of practices, including highlighting, rereading repeatedly, and cramming (Kornell & Bjork, 2007; Hartwig & Dunlosky, 2012; Karpicke et al., 2009).

Given the diversity of students’ approaches, numerous studies have examined the effectiveness of different learning strategies (e.g., Karpicke et al., 2009; Bartoszewski & Gurung, 2015; Hattie & Donoghue, 2018). Highlighting, for instance, is a strategy most students have reported using (Gurung et al., 2010). Thus, many researchers have examined the effect of highlighting/underlining on retention and comprehension (e.g., Hoon, 1974; Ponce et al., 2022). For example, Fowler and Barker (1974) had participants read articles about boredom and city life. Participants were divided into four groups: those who actively highlighted the text, those who passively read the text highlighted by peers, those who read pre-highlighted text by experimenters, and those who read the text without highlighting. One week later, participants took a 54-item multiple-choice test on the material. Participants across the conditions did not significantly differ in their overall test scores, suggesting that, at a minimum, the act of highlighting brought no extra value for learning above and beyond the act of reading (Dunlosky et al., 2013, but also see Ponce et al., 2022 for an alternative view, particularly when highlighting is combined with other practices).

Researchers have likewise examined the impact of numerous learning strategies that students rarely come to utilize on their own. For example, Pan et al. (2025) examined the benefits of interleaved practice in second language (L2) grammar learning by having participants practice verb conjugations and tense identification in Spanish and French using either an interleaved or blocked schedule. Participants in the interleaved condition alternated between tenses during their practice sessions, while participants in the blocked condition practiced one tense at a time. Following the practice sessions, participants’ retention and application of the grammar were tested using a grammaticality judgment test where participants evaluated sentences for correct grammar use. The results showed that participants in the interleaved condition outperformed those in the blocked condition on verb conjugation, tense identification, and language identification tasks. Similarly, numerous studies have now demonstrated that active retrieval-based learning strategies (e.g., testing oneself) produce better learning than more passive approaches (e.g., re-reading) (Roediger & Butler, 2011; Roediger & Karpicke, 2006). For instance, Butler and Roediger (2007) demonstrated that active retrieval practice after lectures significantly improved material retention compared to passively reviewing summaries. Likewise, Bartoszewski and Gurung (2015) found that students who employed practice testing and self-explanation achieved significantly better outcomes on exams compared to those who relied on summarizing or highlighting.

Given that there is now a sizable body of research focused on the effectiveness of many different learning strategies, this has enabled the use of systematic review methods to assess the impact of these learning strategies across various contexts. In their systematic review, Dunlosky et al. (2013) revealed that strategies that promote active engagement with the material, such as practice testing, interleaved practice, and distributed practice, consistently enhance comprehension and retention. In contrast, strategies that encourage less active engagement, like summarizing, highlighting, and rereading, often produce limited benefits.

Researchers have also examined whether students are aware of which strategies are the most effective. Studies surveying college students about their learning strategies revealed that students commonly rely on ineffective approaches such as rereading, highlighting and massing practice (Kornell & Bjork, 2007; Karpicke et al., 2009; McCabe, 2011; Witherby et al., 2025). For example, Kornell and Bjork (2007) found that students largely prioritized urgent deadlines, with 59% studying whatever was due soonest (cramming) and only 11% studying in advance (distributing practice). This result is mirrored by work by Hartwig and Dunlosky (2012), who found that 83.6% of students reported massing their study sessions, and 66% reported rereading (both of which are ineffective strategies). Even when students use strategies like self-testing, many employ self-testing primarily to monitor their understanding rather than enhance their learning.

These findings highlight the need for approaches that could reduce the extent to which students utilize ineffective learning strategies and increase the extent to which they adopt more effective learning strategies. Research has shown that many students engage in less effective practices due to misconceptions about their effectiveness or a lack of awareness of more efficient alternatives (Hartwig & Dunlosky, 2012; Karpicke et al., 2009; McCabe, 2011). Consequently, most studies have focused on addressing these misconceptions through interventions designed to improve students’ understanding of evidence-based learning strategies via one of two main approaches.

The first general approach is instruction, where students are explicitly taught about the effectiveness of learning strategies through videos, lectures, or course modules (Ariel & Karpicke, 2018; Cathey et al., 2016). This method aims to enhance students’ awareness and understanding of evidence-based practices. Studies of this type have produced somewhat mixed results with respect to their impact on changing students’ knowledge and subsequent behaviors (Dembo & Seli, 2004; Foerst et al., 2017; McCabe, 2011). In a more positive direction, Brown-Kramer (2022) had students read one of four empirical articles on learning strategies—distributed practice, rereading, practice testing, or forming mental images—and write a paper to summarize, analyze, and apply the findings. Over the semester, students reported increased use of effective strategies such as practice testing and interleaved practice, while reducing their reliance on less effective strategies like highlighting and summarization. In the less positive direction, Cathey et al. (2016) embedded a midterm study skills training session into an Introductory Psychology course. They presented participants with videos focusing on effective study strategies and common misconceptions about learning. Although students who attended the intervention gained greater insight into their study strategy weaknesses and rated the session as helpful and effective, the training did not significantly change their study time or approach to learning. In sum, it appears that pure instruction-based interventions may produce slight shifts in beliefs, but it remains unclear how much they change learning behaviors, as instruction alone in particular often fails to produce long-term behavioral changes (Balch, 2001; Dembo & Seli, 2004; Foerst et al., 2017).

The second general approach is to provide direct experience by having students actively engage with effective learning strategies. This method aims to have students practice strategies like practice testing and interleaving to help them understand the benefits of these strategies (Einstein et al., 2012). Similarly to the instruction approach, this approach also produced mixed results (DeWinstanley & Bjork, 2004; Gurung & Burns, 2019). For instance, in the positive direction, Balch (2006) provided students with the opportunity to experience distributed versus massed practice of word pairs in a classroom experiment. Students performed better with the distributed pairs, and a follow-up evaluation revealed that they reported a slight shift in their perception and study habits toward using distributed practice. While research indicates that engaging with more effective strategies may encourage positive behavioral changes, individuals often fail to recognize what is beneficial because they misinterpret their experiences. For instance, Pan and Rivers (2023) had participants experience pretesting across multiple cycles and demonstrated that participants had better learning outcomes. However, their perspective of the strategy only changed when they were provided with external guidance that highlighted their improved performance with pretesting. Similarly, Yan et al. (2016) showed that, even after participants benefited from interleaving during category learning, many still believed blocking to be more effective. Only when participants’ attention was explicitly directed to the connection between their performance and the study schedule did their beliefs begin to shift—highlighting the challenge of correcting metacognitive illusions through experience alone.

Both instruction and experience-based approaches have clear benefits but often fail to produce significant changes in understanding and behavior when used alone. Instruction increases awareness but rarely leads to lasting behavioral change, while experience provides opportunities for implementation, but rarely produces the understanding without explicit guidance. Yüksel et al. (2024) combined these two approaches by integrating direct instruction with experience. They took advantage of a natural experimental intervention in a college classroom where one instructor of Introduction to Psychology both taught students about effective strategies in a full dedicated lecture and also embedded these practices into the course structure. For instance, students learned about the benefits of spacing learning sessions over massed practice (i.e., cramming) as part of the lecture on effective learning practices in academia. They also took cumulative quizzes throughout the semester designed to make them space their study sessions (with the instructor clearly tying the practice to the explicit instruction). Yüksel et al. (2024) demonstrated that this combination of direct experience and direct practice produced notable gains in understanding and use of learning strategies in accordance with the empirical evidence around effectiveness.

An open question is whether these improvements persist beyond the intervention. Few studies have examined the long-term durability of these changes over extended periods, such as a semester or longer, to determine whether these strategies continue to influence behavior and performance (e.g., Usdansky et al., 2024; Brown-Kramer, 2022). However, the small number of studies that have done so suggest that producing long-term changes in behavior is likely possible. For instance, Brown-Kramer (2022) examined the long-term effects of an intervention in which students completed term papers on learning strategies. The study compared a control semester to one where students wrote about varied strategies. Results showed that students who engaged with effective strategies, such as practice testing, demonstrated improved strategy use and better course performance in the following semester.

To further assess the extent to which long-term changes in beliefs and use of effective and ineffective learning strategies are feasible, the current research examines the long-term impact of the intervention described above in Yüksel et al. (2024), which utilized both explicit instruction about both effective and ineffective learning strategies as well as direct experience with effective strategies. In Study 1, we assessed whether participants maintained changes in their beliefs about learning strategies and their effectiveness six months after the intervention. In Study 2, we expanded the scope by including students from various academic years by analyzing data from those who enrolled in the intervention section of the course over a five-year period. To foreshadow the results, we found that reported behaviors (i.e., the learning strategies that students actually used) often belied their knowledge. Indeed, students strongly retained their understanding of which approaches were likely to be more or less effective, but nonetheless frequently reported utilizing strategies they knew were less effective. As such, we also gathered information via open-ended questions with the specific goal of understanding the obstacles that the students perceived were preventing them from using more effective learning strategies.

## 2. Study 1

The current work builds upon a previously published intervention study (Yüksel et al., 2024) in which students in one section of an Introduction to Psychology course received instruction on and experience with effective practices in learning (intervention group), while students in other sections did not (business-as-usual group). While Yüksel et al. (2024) only examined the impact of the intervention shortly after the conclusion of the intervention, here we collected long-term follow-up data to determine whether the changes in beliefs about, and use of, effective learning strategies that were observed directly post-intervention persisted long-term. Specifically, for Study 1, the new data corresponds to the long-term follow-up assessments collected approximately six months after the intervention. The corresponding subset of pre-intervention and post-intervention data (the full set of which was reported in Yüksel et al., 2024) are included here to provide a clear point of comparison. In the intervention condition, surveys were administered at three time points labeled relative to the intervention period: “pre-intervention” (before the intervention), “post-intervention” (at the end of the course), and “long-term” (approximately six months later). We administered surveys in the business-as-usual condition at times comparable to the administration of surveys in the intervention condition; that is, the pre-intervention survey occurred before the intervention period, the post-intervention survey at the end of the course, and the long-term follow-up six months later.

### 2.1. Method

#### 2.1.1. Participants

We reached out to the 316 (intervention: 118, business-as-usual: 198) participants who had taken part in the intervention portion of Yüksel et al. (2024). In total, 62 participants agreed to participate, of which 54 (38 women, *Mage* = 19.11) had pre-intervention data available. All participants provided consent in accordance with an approved IRB protocol, and those who participated in the study received a USD 5 Amazon gift card.

#### 2.1.2. Procedure

While the focus of the current manuscript is on the long-term persistence of changes in learning strategy use as a function of the interventions utilized in Yüksel et al. (2024), here we briefly describe the full set of methods (see Yüksel et al., 2024, for a complete description of all procedures).

**Intervention:** The intervention group (*N* = 15) was one section of Introduction to Psychology that included a dedicated module aimed at teaching effective learning strategies. This module highlighted evidence-based effective learning practices, such as spacing study sessions over time rather than cramming, interleaving topics instead of focusing on one at a time (blocking) and employing active learning techniques like retrieving information rather than passively reading it. These strategies were taught and framed in terms of their benefits for long-term learning. The instruction was designed to promote active engagement with the material, fostering deep processing over passive learning. In addition to covering effective learning strategies, the module also mentioned commonly used learning strategies with limited effectiveness, such as highlighting text passively, rereading, copying notes, summarizing, cramming, and using keyword mnemonics.

To support students’ understanding of these strategies, the course also integrated the general principles into the semester’s structure. This included cumulative weekly quizzes to encourage interleaving and spacing, varied seating arrangements to support contextual variability, and activities like generating quiz questions to facilitate deeper processing. Students not only practiced these strategies but also learned the rationale behind them. For instance, they were taught that cumulative exams are designed to promote interleaving and spacing in their study routines.

**Business-as-Usual:** The business-as-usual sections of Introduction to Psychology also covered some of the materials covered in the intervention section, but in a far less extensive fashion. Similarly, while a few strategies were implemented to some extent in the business-as-usual sections (e.g., weekly non-cumulative quizzes), these practices were less comprehensive than the intervention section. Because there were no significant differences in either perceived effectiveness or reported frequency of use among any of the business-as-usual courses (*N* = 39), these were combined into a single group.

#### 2.1.3. Assessments

For each of the 16 different learning strategies—rereading, group studying, cramming, highlighting, using flashcards, thinking of real-life examples, note-taking, practice tests, looking over notes, copying notes, summarizing, creating an outline, studying in different places, spacing, self-explanation, and interleaving—participants were asked to report the extent to which they used the given strategies and whether they viewed the strategies as effective/ineffective. Specifically, participants rated the frequency of use and perceived effectiveness of each of the learning strategies on a 1–10 scale, with 1 indicating “not at all” and 10 indicating “very frequently” or “very effective”. For frequency of use, participants responded to the prompt: “How frequently have you been using each of the following studying methods this semester?” For perceived effectiveness, they answered: “How would you rate the effectiveness of each studying method?” (see Appendix C for an example, and the questionnaires folder on OSF for the full survey).

The participants’ responses were then matched with their responses taken prior to the intervention (pre-test) and/or directly after the intervention (post-test). Four of the sixteen learning strategies were added for this study and thus have no corresponding pre-test/post-test data (studying in different places, spacing, self-explanation, and interleaving). These four strategies were assessed via scenario-based questions in Yüksel et al. (2024); however, to reduce participant burden in the current work, these were switched to simple frequency/effectiveness measures here.

We also included several open-ended questions to gain deeper insights into participants’ study behaviors and potential barriers to adopting effective strategies. These questions asked participants to list the classes they were taking that semester (e.g., “Please list all of the classes that you’re taking this semester”), describe their study habits, indicate any changes in study habits compared to the previous semester, and identify strategies they had not used but would like to. Additionally, we asked them to reflect on the barriers preventing them from using the strategies they perceive as effective ones (e.g., “What are the barriers to using study habits you have not used this semester but would like to use?”).

All assessments were conducted online via Qualtrics. During the pre-test and post-test phases, participants completed the measures in the following order: perceived effectiveness, reported frequency of use, and scenario-based questions. The items and scenarios were presented in a randomized order. However, the scenarios are not relevant to the current study, as they were not included in the long-term follow-up. In the long-term follow-up, participants completed open-ended questions first, followed by the perceived effectiveness and reported frequency of use measures. The order of items within each measure was randomized.

### 2.2. Results

All data analyses were performed through R (R Core Team, 2017). The data files and script folders, where all data analyses are performed, are available on the Open Science Framework (OSF, https://osf.io/kcduz/?view_only=8ba58424b68743c9a39a6e7b66c34516 accessed on 24 April 2025).

The dependent variables were the perceived effectiveness and reported frequency of use of effective and ineffective strategies. For the main analyses of perceived effectiveness and reported frequency of use, we calculated two separate aggregate scores by grouping learning strategies into effective and ineffective learning strategies. We followed the classification from Dunlosky et al.’s (2013) systematic review and the related literature to define these categories. Effective strategies included taking tests, using flashcards, applying real-life examples, creating outlines, group study, studying in different places, spacing the study schedules, self-explanation, and interleaving the study material. While such a binary (effective/ineffective) classification scheme is not capable of capturing various nuances (e.g., the extent to which certain strategies are effective can depend on the manner in which they are employed), it was utilized in the current work for several reasons. First, we used the classification from Dunlosky et al. (2013) as a framework for presenting learning strategies to students. Indeed, this categorization allowed for a method that was independent of the research team. That is, what was described as effective was based not on instructor’s beliefs, but rather the science of learning. Finally, this categorization also allows for a direct match with previously published work using pre-registered analyses (Yüksel et al., 2024; note the current work was not pre-registered, but did directly follow the previous work that was). For readers interested in possible alternative schemes the data fully broken out by individual strategies are available in the Appendix A.1.

Because we included four new effective learning strategies in this follow-up work, we calculated two scores for effective learning strategies: one for the combination of the 12 learning strategies utilized in Yüksel et al. (2024) and one for the full set of 16 strategies included in the current study. Then, we confirmed that these two measures were not significantly different from each other, either for perceived effectiveness (*t*(53) = 1.55, *p* = 0.13, 95% CI [−0.05, 0.36]) or for reported frequency of use ratings (*t*(53) = −0.33, *p* = 0.74, 95% CI [−0.23, 0.16]). Ineffective strategies consisted of highlighting, copying notes, copying the textbook, cramming, rereading, and summarizing. Higher scores reflected greater understanding, perceived effectiveness, and frequency of use.

#### 2.2.1. Pre-Intervention Comparison of Groups

We first assessed whether there were any significant differences between the intervention group and the business-as-usual group prior to our analyses to ensure that any observed differences emerged after the intervention. At pre-intervention, participants did not differ significantly on any outcome: perceived effectiveness of effective, F(1,43) = 1.19, *p* = 0.28, or ineffective strategies, F(1,43) = 0.92, *p* = 0.34; reported frequency of use of effective, F(1,43) = 0.73, *p* = 0.78, and ineffective strategies, F(1,43) = 0.36, *p* = 0.54. Thus, there is no evidence to suggest that the groups differed before beginning the study.

#### 2.2.2. Use and Perceived Effectiveness of Effective Learning Strategies

To determine whether enrollment in the intervention group was associated with perceived effectiveness and use of learning strategies compared to the business-as-usual group, we fitted a series of linear mixed-effects models on four dependent variables: perceived effectiveness of effective strategies, perceived effectiveness of ineffective strategies, reported frequency of use of effective strategies, and reported frequency of use of ineffective strategies. We regressed the dependent variables of interests on group (contrast coded: “−0.5” for business-as-usual and “0.5” for intervention), time (treated as a categorical factor: pre-intervention vs. post-intervention, vs. long-term follow-up) and their interaction, controlling for the random effects of time.

**Perceived Effectiveness of Learning Strategies.** Two linear mixed effect models were conducted to examine the effects of time, group, and their interaction on the perceived effectiveness of effective and ineffective learning strategies, controlling for random effects of time.

**Figure 1:**
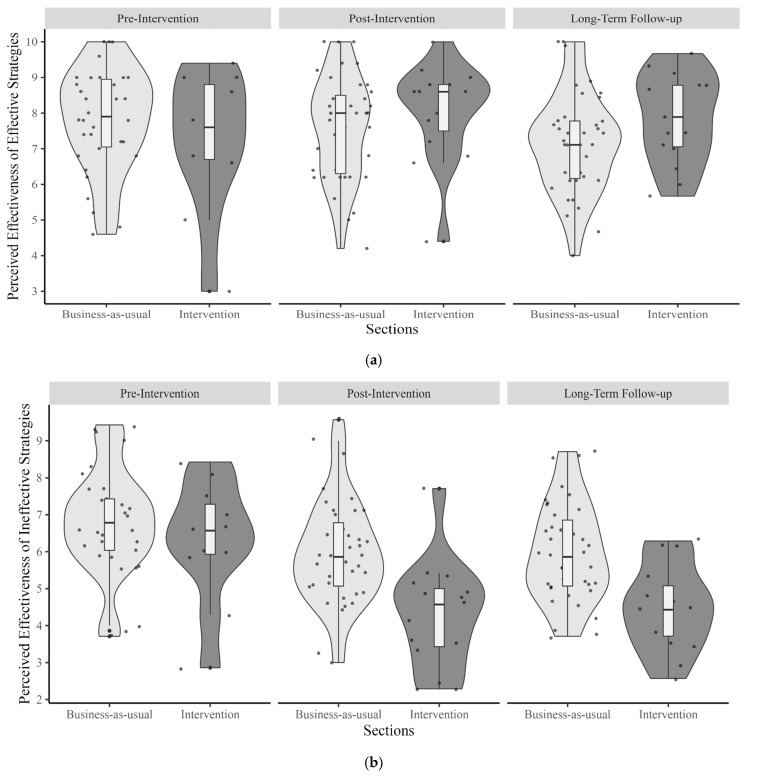
(**a**) Pre-intervention, post-intervention, and follow-up ratings of perceived effectiveness of effective strategies across groups. (**b**) Pre-intervention, post-intervention, and follow-up ratings of perceived effectiveness of ineffective strategies across groups.

For effective learning strategies, the results revealed no significant main effect of time. Perceived effectiveness did not differ significantly between the post-intervention (*b* = 0.29, *SE* = 0.41, *t* = 0.70, *p* = 0.48) or the long-term follow-up (*b* = −0.03, *SE* = 0.41, *t* = −0.07, *p* = 0.95) compared to the pre-intervention phase. Similarly, the main effect of group, intervention or business-as-usual, was not significant (*b* = −0.59, *SE* = 0.50, *t* = −1.18, *p* = 0.24). An interaction between time and group was significant: students in the intervention group (*M* = 7.86) reported higher perceived effectiveness for effective learning strategies than those in the business-as-usual group (*M* = 7.14) during the long-term follow-up (*b* = 1.31, *SE* = 0.67, *t* = 1.98, *p* = 0.04), whereas the groups did not significantly differ during post-intervention (*Mintervention* = 8.05, *Mbusiness-as-usual* = 7.59) (*b* = 1.05, *SE* = 0.67, *t* = 1.58, *p* = 0.117) compared to the pre-intervention (*Mintervention* = 7.24, *Mbusiness-as-usual* = 7.83) (Table 1, Figure 1a).

For ineffective learning strategies, the main effect of time was not significant; however, we observed a trend. Students reported lower perceived effectiveness of ineffective strategies during the post-intervention (*M* = 5.50) (*b* = −1.43, *SE* = 0.76, *t* = −1.87, *p* = 0.06) and long-term follow-up (*M* = 5.55) (*b* = −1.33, *SE* = 0.76, *t* = −1.74, *p* = 0.08) compared to the pre-intervention (*M* = 6.68). The main effect of the group was not significant (*b* = −0.49, *SE* = 0.48, *t* = −1.01, *p* = 0.312). The interaction between time and group was also not significant. Overall, students in the intervention group (*M* = 4.28) reported lower perceived effectiveness than those in the business-as-usual group (*M* = 5.97) during post-intervention (*b* = −1.20, *SE* = 0.64, *t* = −1.88, *p* = 0.062), whereas the groups made similar ratings in the pre-intervention phase (*Mintervention* = 6.31, *Mbusiness-as-usual* = 6.79) and the long-term follow-up (*Mintervention* = 4.48, *Mbusiness-as-usual* = 5.95) (Table 2, Figure 1b).

**Reported Frequency of Use of Learning Strategies**. As above, two linear mixed effect models were constructed to examine the effects of time, group, and their interaction on the reported frequency of use of effective and ineffective learning strategies.

**Figure 2:**
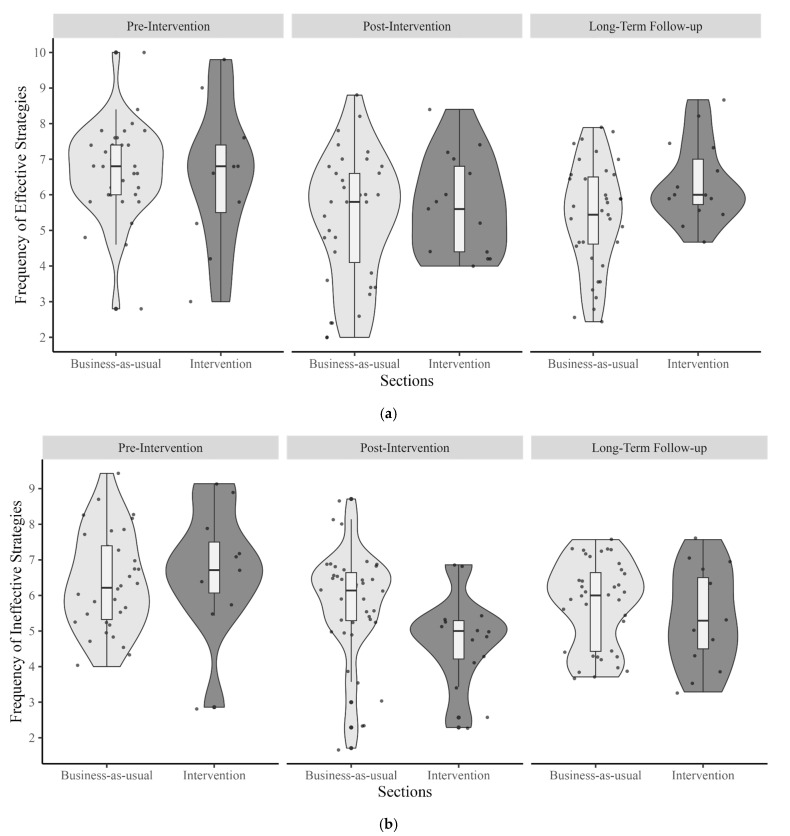
(**a**) Pre-intervention, post-intervention, and follow-up ratings of frequency of use of effective learning strategies across groups. (**b**) Pre-intervention, post-intervention, and follow-up ratings of frequency of use of ineffective learning strategies across groups.

For effective learning strategies, the results revealed a significant main effect of time. Participants reported significantly lower frequency of effective strategy use in the post-intervention (*M* = 5.44) (*b* = −1.08, *SE* = 0.35, *t* = −3.11, *p* = 0.004) and in the long-term follow-up phase (*M* = 5.65) (*b* = −0.75, *SE* = 0.35, *t* = −2.15, *p* = 0.03) compared to the pre-intervention (*M* = 6.65). We did not find a significant main effect of group (*b* = −0.14, *SE* = 0.52, *t* = −0.26, *p* = 0.80). Moreover, the interaction between time and group was not significant such that students’ ratings did not differ across groups during the post-intervention (*b* = 0.54, *SE* = 0.70, *t* = 0.78, *p* = 0.44) or long-term follow-up (*b* = 1.09, *SE* = 0.70, *t* = 1.56, *p* = 0.12) compared to the pre-intervention (Figure 2a).

Similarly, for ineffective learning strategies, there was no significant difference in students’ ratings across time points. However, a trend was observed, such that students’ ratings for frequency of ineffective strategy use were lower in the post-intervention (*M* = 5.46) (*b* = −1.31, *SE* = 0.67, *t* = −1.93, *p* = 0.055) and in the long-term follow-up (*M* = 5.67) (*b* = −0.96, *SE* = 0.67, *t* = −1.43, *p* = 0.15) compared to their ratings in the pre-intervention (*M* = 6.47). No significant main effect of group was found (*b* = 0.31, *SE* = 0.49, *t* = 0.63, *p* = 0.53). There was an interaction effect between time and group: students in the intervention group reported less frequency of use of ineffective learning strategies during the post-intervention compared to the pre-intervention (*b* = −1.30, *SE* = 0.64, *t* = −2.02, *p* = 0.04); however, their ratings were not significantly different across the long-term follow-up (*b* = −0.69, *SE* = 0.64, *t* = −1.07, *p* = 0.28) and the pre-intervention (Figure 2b).

While we calculated aggregate scores for effective and ineffective strategies to facilitate interpretation in our main analyses, we also included detailed analyses of each individual learning strategy in Appendix A.1, Table A1 and Table A2. Additionally, due to the low number of responses to open-ended questions, we report them descriptively in Appendix A.2, Table A3 and Table A4.

## 3. Study 2

In Study 1, we observed that some of the changes in understanding about effective/ineffective learning strategies observed immediately after the intervention persisted six months after the intervention. However, there was no sustained change in the reported frequency of use of learning strategies. Study 2 was conducted to further investigate whether the effects on understanding remained consistent over an extended period and whether they eventually contributed to any meaningful changes in the use of effective or ineffective strategies. Moreover, we aimed to expand our participant pool in Study 2; we reached out to all students who had enrolled in the Introduction to Psychology course at University of Wisconsin-Madison during the past five years. By gathering responses from students across different sections and academic years, we could examine if there were any differences in understanding or use of learning strategies across sections that might emerge over time.

### 3.1. Method

#### 3.1.1. Participants

We contacted a total of 12,611 (intervention: 1454, business-as-usual: 11,156) students who enrolled in various sections of Introduction to Psychology at University of Wisconsin-Madison over the past five years. In total, 117 (90 women, *Mage* = 20.87) participants opted to take part in the study and were entered into a USD 100 lottery for their participation. Among these participants, 102 (77 women, *Mage* = 20.84) completed the full survey and passed the attention check question that was asked to see if participants were properly reading all the questions in the survey. Data from these 102 participants were used in the quantitative analyses. Of those, 68 were previously enrolled in one of the business-as-usual sections and 34 were enrolled in the intervention section. However, for the open-ended questions, we included responses from all 117 (80 business-as-usual, 37 intervention) participants who answered, regardless of whether they completed the full survey or passed the attention check, in order to retain all available qualitative data. Notably, nine of these participants were enrolled in the same semester as the participants in Study 1 (Spring 2021, see Appendix B, Table A5 for full list), and only one participant took part in both studies (the inclusion/exclusion of this participant made no difference in the reported outcome and thus they were included to maximize the size of the dataset).

#### 3.1.2. Assessments

**Figure A1:**
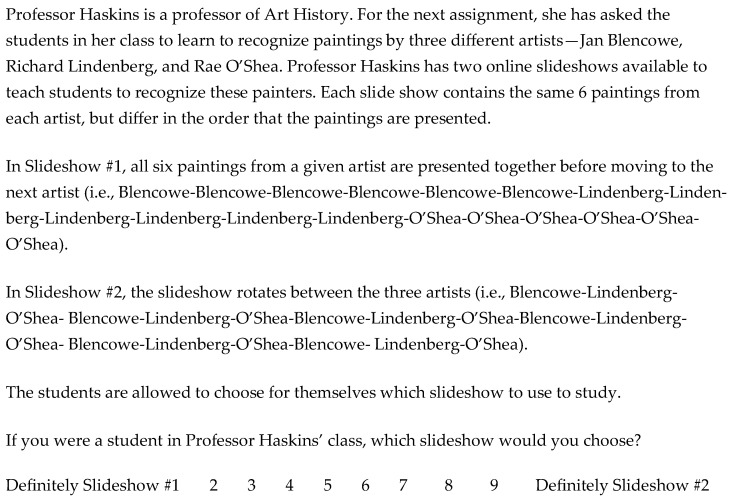
Example scenario question (interleaving vs. blocking).

**Figure A2:**
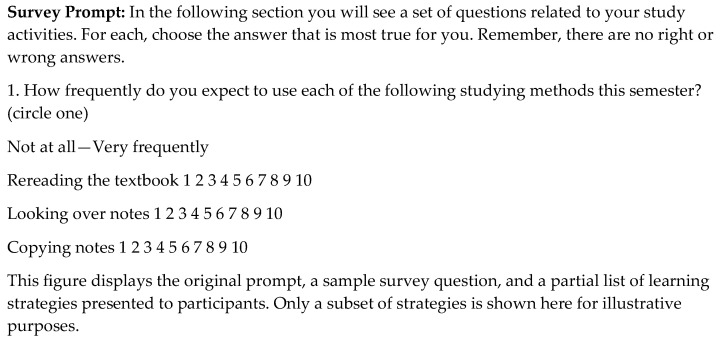
Example Survey Prompt, Question, and Sample Learning Strategies.

Participants were asked to complete the same set of questionnaires used in Study 1, which included ratings of the perceived effectiveness and reported frequency of use of both ineffective and effective learning strategies. All assessments were administered post-intervention. Differently from Study 1, Study 2 also included scenario-based questions from Yüksel et al. (2024) that were designed to assess participants’ ability to apply learning strategies in context (see Appendix C, Figure A1 and Figure A2 for examples and OSF for the full set). Additionally, participants answered open-ended questions about their general study behaviors and described any obstacles they encountered when attempting to use effective strategies.

Participants responded to a series of open-ended questions designed to assess their study habits, learning strategies, understanding of learning strategies, and perceived barriers. These questions were adapted from Study 1, but this study aimed to examine these areas in greater detail. Participants were asked to describe their current study routines and habits for their classes and exams, assess whether their study habits and learning strategies were effective for their learning, and provide explanations for their evaluations (e.g., Do you think that the study habits/learning strategies you are using are the effective ones for your learning needs? Why/not?). They also reflected on how their study routines and habits had changed over time in college and listed any new study habits or learning strategies they were using (e.g., How have your study routines/habits changed over the course of your time in college?). Additionally, participants identified study habits or learning strategies they had not yet implemented but would like to, along with the barriers preventing them from doing so (e.g., Are there any study habits/learning strategies that you have not used, but would like to implement? Please list all of the habits/strategies that you can think of; What are the barriers to using study habits/learning strategies you have not used this semester, but you would like to use?). Finally, they elaborated on the obstacles preventing them from adopting strategies they had heard or learned were effective but had not yet utilized (e.g., What are the barriers preventing you from using study habits/learning strategies that you have heard/learned are effective but have not yet used?).

We also included the list of scenarios utilized in Yüksel et al. (2024) which served as a measure of participants’ understanding of learning strategies. These scenarios were included in the current study to evaluate whether, after a significant amount of time, participants could identify effective and ineffective strategies in hypothetical contexts. Participants were presented with various scenarios and asked to choose which strategy they would most likely use if they were the student in the given situation. For instance, participants were presented with a scenario in which a professor offered two study options to help students learn painting styles by three different artists: one slideshow grouped all paintings by artist, while the other alternated paintings between artists. Participants were asked to choose which slideshow they would prefer to use if they were a student in this class (see more examples from https://osf.io/kcduz/?view_only=8ba58424b68743c9a39a6e7b66c34516 accessed on 24 April 2025).

### 3.2. Results

Consistent with Study 1, we calculated aggregate scores for the perceived effectiveness and reported frequency of use of both effective and ineffective learning strategies. Additionally, we calculated aggregate scores for the scenario questions. For effective learning strategies and scenario outcomes, higher scores reflected a better understanding of effective learning strategies. Conversely, for ineffective learning strategies, lower scores indicated a better understanding, as they demonstrated the ability to recognize and avoid less effective strategies. Following this, we conducted several linear regression analyses where the dependent variables were regressed on the groups (intervention vs. business-as-usual), controlling for the semester (from 2020 Fall to 2024 Spring). We included semester as a control variable rather than conducting semester-level analyses because the number of participants per semester was limited (e.g., some semesters had no participants; see Appendix B, Table A5 for full breakdown of participants by group and semester).

#### 3.2.1. Quantitative Assessments

**Figure 3:**
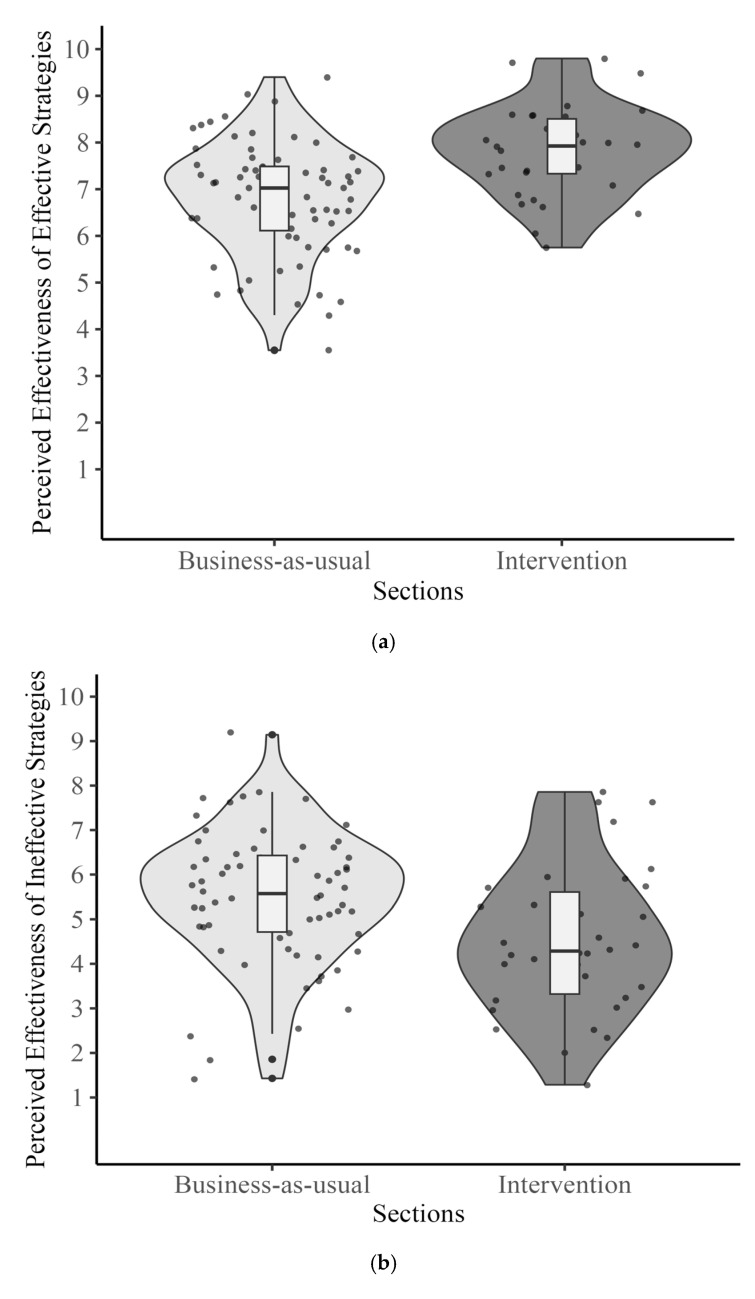
(**a**) Ratings of perceived effectiveness of effective strategies. (**b**) Ratings of perceived effectiveness of ineffective strategies.

**Perceived Effectiveness of Learning Strategies**. The analysis revealed that the perceived effectiveness of effective strategies was significantly higher for the intervention group, *b* = 1.36, *t*(91) = 4.38, *p <* 0.001, with means of 7.83 and 6.79, respectively (see Table 3, Figure 3a). In contrast, while the effect on ineffective strategies was numerically the same magnitude as for effective strategies (intervention: *M* = 4.50; business-as-usual: *M* = 5.49), this did not reach significance, *b* = −0.46, *t*(91) = −1.15, *p* = 0.25 (see Table 3, Figure 3b).

**Figure 4:**
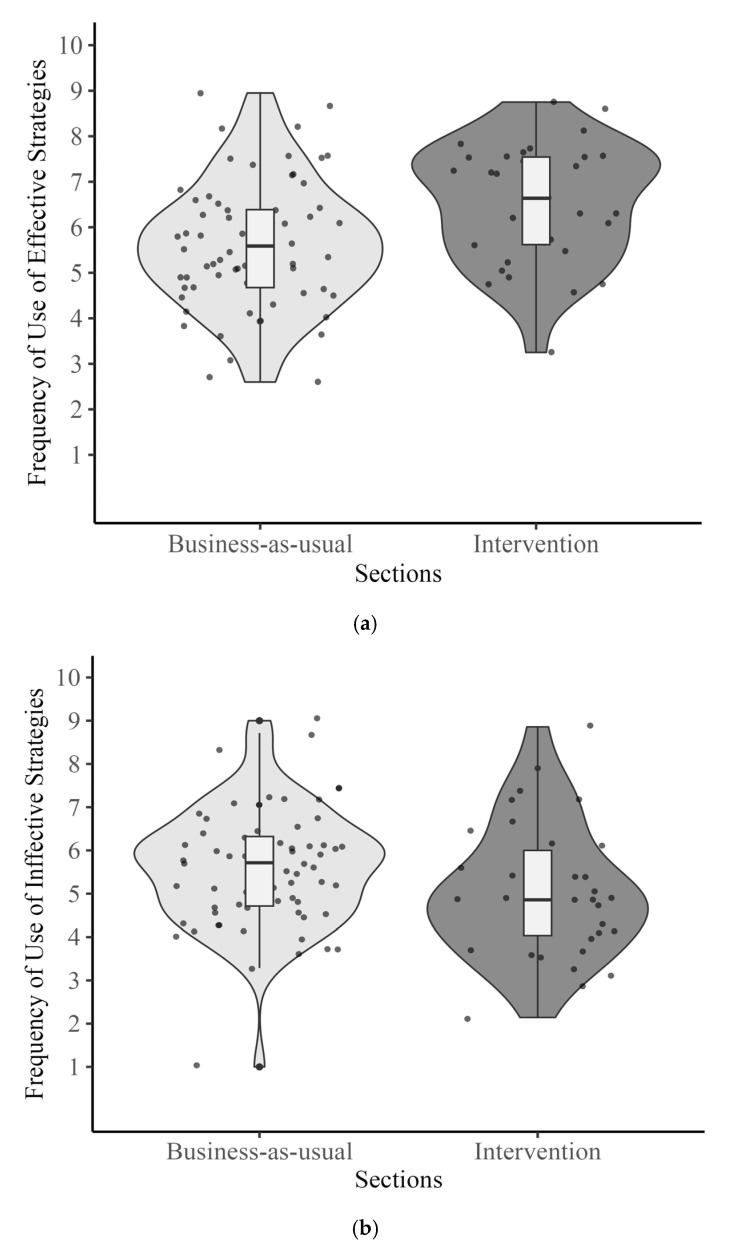
(**a**) Ratings of reported frequency of use of effective strategies. (**b**) Ratings of reported frequency of use of ineffective strategies.

**Reported Frequency of Use of Learning Strategies.** The results demonstrated that there was a significant difference between groups for the reported frequency of effective strategy use, *b* = 0.95, *t*(91) = 2.57, *p* = 0.01, such that students in the intervention group (*M* = 6.51) reported higher ratings compared to those in the business-as-usual group (*M* = 5.62) (see Figure 4a). However, the frequency of using ineffective strategies showed no significant difference between the groups (*Mintervention* = 5.04, *Mbusiness-as-usual* = 5.61), *b* = −0.13, *t*(91) = −0.34, *p* = 0.73 (see Figure 4b).

**Figure 5:**
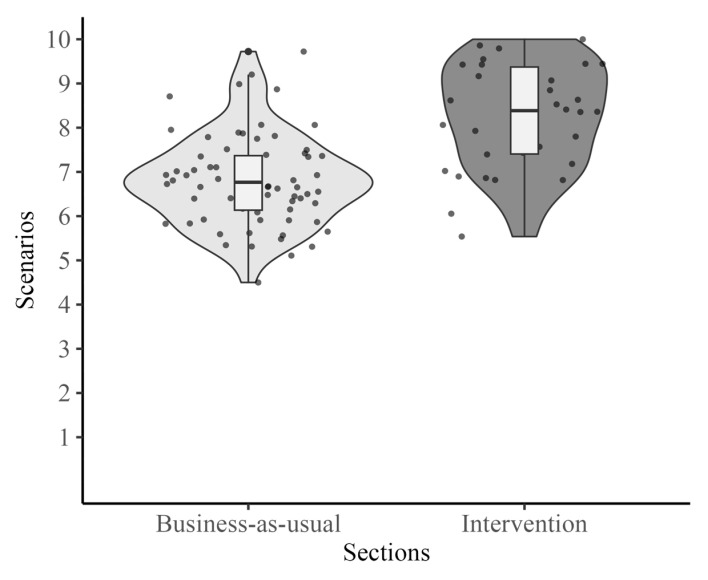
Scenarios ratings.

**Scenarios.** A linear regression analysis revealed a significant difference between groups in scenario scores, *b* = 1.56, *t*(91) = 5.52, *p <* 0.001. Students in the intervention group had higher scores (*M* = 8.28) than those in the business-as-usual group (*M* = 6.81) (see Figure 5).

Although our main analyses relied on aggregate scores for effective and ineffective strategies, we also provided item-level analyses for each individual learning strategy in Appendix B.2, Table A6 and Table A7.

#### 3.2.2. Open-Ended Questions

Students’ responses to open-ended questions were coded by two independent coders who were unaware of the study’s predictions or participants’ groups (intervention vs. business-as-usual). We created three main coding schemes including learning strategies mentioned, reasons strategies were perceived as effective or ineffective, and obstacles (see OSF link above for the coding schemes). The coding schemes were developed collaboratively by the first independent coder (outside of the research team, but aware of the general research questions) and the experimenter (E.Y.) through discussions of approximately 15 responses for each question. Subsequently, both coders applied the finalized coding scheme to the entire dataset. Given the fully open-ended nature of the responses, all relevant matches (e.g., learning strategies, obstacles) were coded. Any discrepancy in the listed responses between coders was counted as a mismatch. Using this method, an inter-rater reliability of 87% was achieved.

The main disagreement was related to the definition of cramming versus spacing as a learning strategy. One coder initially categorized studying 4–7 days before the exam as studying in advance (and thus a “spacing” strategy), whereas the other coded this as cramming. After discussion, it was decided to set the cutoff for defining cramming as less than 7 days before an exam. After finalizing the coding based on the final discussion, the number of participants in each condition for each response was counted. The number of participants (and percentages) reported below reflect only those who responded to each specific question. Percentages are calculated by dividing the number of participants who mentioned a given strategy, reason, or obstacle by the total number of participants who answered that question. Because some participants skipped certain questions (e.g., did not report obstacles but explained why their strategies were effective), the denominators vary. Participants could be counted in multiple categories if they mentioned more than one strategy, reason, or obstacle. For instance, if a participant stated that they regularly study for their exams daily or weekly and also study for long hours the night before an exam, they would be coded both as using spacing (i.e., distributed practice) and as cramming.

**Can You Describe Your Current Study Routine and Habits for Your Current Classes and Exams?** Students provided detailed information about their current study routines (see Table 4). They listed many learning strategies within the spectrum of both effective and ineffective approaches. They also reported several learning strategies that have not been extensively studied in the literature.

Among the more commonly endorsed effective strategies were retrieval practice and spacing. However, most of the reported spacing was due to weekly quizzes and assignments rather than intentionally spacing study sessions. Among the less active strategies, rereading learning materials—such as textbooks, notes, and slides—was the most commonly reported strategy among both groups (intervention and business as usual). Cramming, defined as heavy studying immediately (~7 days) before exams, was also frequently mentioned by students in both the business-as-usual and intervention groups. Two other less active learning strategies mentioned by students were rewriting notes and creating outlines of the key points. Additionally, a notable number of students expressed efforts to improve their time management (e.g., use of planning tools like Pomodoro) to organize their study schedules. Some participants described learning strategies that have not been widely studied. For example, listening to audiobooks or watching recorded class videos.

**Do You Think That the Study Habits/Strategies You Are Using Are Effective?** When students were asked to judge if their learning strategies were effective, the majority of the students (77%) reported that they believed their learning strategies were effective, whereas only a handful of students were unsure, and only a few believed they used ineffective approaches. These ratings were consistent across the groups (see Table 5).

Among those who believed their strategies were effective, the majority reported rereading, cramming, and copying notes—approaches that are generally less aligned with long-term learning goals. In contrast, a number of students mentioned strategies like retrieval practice and spacing, which are supported by studies for promoting long-term retention and understanding.

**Why/Why Not (In)Effective?** As a follow-up to students’ judgments of the effectiveness of their learning strategies, they were asked to elaborate on their reasoning. Those who rated their strategies as effective were asked why they found them effective, while those who rated them as ineffective were asked why they believed the strategies were ineffective. Students who were unsure were asked both questions to investigate which aspects they considered effective and which they perceived as ineffective.

When asked why their strategies were ineffective, some students stated that they were aware of more effective alternatives but did not elaborate on why they believed their own strategies were ineffective. A few students attributed their continued reliance on these strategies to procrastination and a lack of organization, while others cited limited access to information about better approaches (see Table 6).

When asked why their strategies were effective, achieving good grades was the most frequent reason among both groups. Additionally, the majority of students stated that they believe their methods helped them learn effectively, while some of these students specifically emphasized that their strategies improved understanding rather than memorization. Also, some mentioned their strategies provide a good representation of exams and help them save time (see Table 7).

**Changes in Study Routines Over Time.** To capture changes in students’ approaches over time, two questions were utilized: “How have your study routines/habits changed over the course of your time in college?” and “What are the new study habits/learning strategies you are using?” Because students frequently responded similarly to both questions, we combined their responses (see Table 8).

Many students reported an increased use of retrieval practice as a learning strategy over time, a trend observed consistently across both groups. Additionally, improvements in time management were commonly mentioned, with several students indicating that they had developed better organizational skills and planned to continue working on their time management skills. Rereading learning materials, such as textbooks and notes, was another strategy that students reported both using and intending to use more frequently. Less commonly mentioned changes included strategies such as spacing and creating outlines of key points in the learning material. Interestingly, some strategies were only mentioned by students in the intervention group, including interleaved practice and self-explanation.

**Obstacles to Adopting New Strategies**. To understand the obstacles students face in adopting effective study strategies, two questions were asked: “What are the barriers to using study habits/learning strategies you have not used this semester but would like to use?” and “What are the barriers preventing you from using study habits/learning strategies that you have heard/learned are effective but have not yet used?” Because students provided similar responses to both questions, their responses to these two questions were combined to summarize the obstacles they reported (see Table 9).

The most commonly cited barrier was lack of time, with many students stating that their packed schedules and competing priorities prevented them from trying more effortful strategies. Another frequently mentioned obstacle was lack of resources, which for many students specifically referred to the unavailability of tools such as retrieval practice tests, which they perceived as beneficial but difficult to access unless provided by instructors. Additionally, a lack of desire to adopt new strategies was widely reported across both groups, with some students feeling unmotivated or believing that changing their study habits was unnecessary.

Other reported barriers included procrastination, which many students attributed to laziness, and uncertainty about effectiveness, with some participants expressing doubt about whether new strategies would actually improve their learning outcomes—particularly their grades. Distractions were another concern, with some students noting that spacing their study sessions made it easier for them to lose focus between intervals, in particular, some students stated that they believed it disrupted their ability to stay in the flow. Lastly, difficulty in changing habits was highlighted as a barrier, as students found it challenging to leave the familiar but less effective learning approaches.

## 4. Discussion

We examined whether an intervention on the effectiveness of learning strategies, consisting of instruction and direct experience with effective learning practices, could lead to long-term shifts in students’ understanding and use of these strategies. Our findings indicated that, compared to the business-as-usual sections of Introduction to Psychology, the intervention led to meaningful improvements in students’ understanding and beliefs about the effectiveness of various learning strategies. Indeed, a key finding of the study was that students in the intervention group not only maintained their increased understanding of effective learning strategies over time but also continued applying them, in contrast to the decline observed in the business-as-usual group. This stability suggests that students who engaged in the intervention were able to internalize and implement at least some of the effective strategies without continued external guidance.

The persistence in students’ understanding and use of effective learning strategies likely stems from the integration of both instruction and experience-based practice. The intervention enhanced students’ knowledge through explicit teaching of the principles underlying effective strategies as well as by ensuring that students utilized many of those effective practices to study the material throughout the semester (e.g., ensuring that they were forced to study material about effective learning practices via active retrieval-based means at timepoints distributed throughout the entirety of the semester, etc.). The intervention thus also helped reinforce that these strategies are effective by providing opportunities to experience them firsthand. This dual approach addressed limitations noted in previous research, which suggests that experience-based approaches may improve behaviors but struggle to enhance understanding without explicit guidance (Pan & Rivers, 2023), and that instruction alone may lead to modest shifts in beliefs but often fails to produce long-term behavioral change (Balch, 2001; Dembo & Seli, 2004; Foerst et al., 2017). This combined approach promoted more enduring changes in students’ knowledge and application of effective learning strategies.

Another notable finding was that a similar pattern was observed for ineffective strategies as well. In the immediate post-intervention period, students improved their understanding of effective strategies and reduced their use of less effective ones. This suggests that the intervention successfully facilitated an initial shift in both knowledge and behavior regarding ineffective strategies. However, while students maintained their improved understanding over time, the changes in behavior did not persist. In the long-term follow-up, some students reverted to their previous habits, and the reduction in reliance on ineffective strategies diminished.

While it is encouraging that students maintained their use of effective strategies over time, the lack of a substantial increase—along with a return to some ineffective strategies—demonstrates the ongoing challenge of translating knowledge into behavioral change. This pattern reflects a well-documented persistent challenge in prior intervention studies: even as students’ understanding of effective strategies grows, they often continue to rely on familiar but ineffective approaches (Ariel & Karpicke, 2018; Brown-Kramer, 2022; Gurung & Burns, 2019; Yüksel et al., 2024). To better understand these challenges, we examined students’ self-reported experiences through open-ended questions. These questions provided additional information on how students conceptualize effective learning and the barriers they encounter when attempting to implement effective strategies.

One critical finding that may play a role in the observed mismatch between knowledge and behavior was with respect to the participants’ stated goals in courses. While most experimental work starts from the premise that students seek to learn, this was not a major goal of our participants. Instead, many students stated that their strategies were effective because they helped them achieve good grades. Their primary goal was performing well on exams and assignments, whereas the intervention aimed to promote changes in learning strategies that support long-term retention (see Soderstrom & Bjork, 2015). This misalignment in goals may make students less likely to implement some of the effective strategies suggested by the intervention. For instance, rereading can be relatively effective for immediate recall (van den Broek et al., 2014), reinforcing the belief that it is a useful strategy, even though it may not support long-term learning as well as strategies like spacing or retrieval practice.

This finding offers useful directions for the design of future studies and interventions, highlighting the importance of addressing the misalignment between students’ goals and long-term learning outcomes. One way to support this shift might be to incorporate goal-setting components into the intervention, explicitly encouraging students to align their study strategies with long-term retention rather than short-term performance. At the same time, instructors play a crucial role in shaping students’ learning priorities. Structuring courses to emphasize conceptual understanding, mastery, and sustained retention, rather than focusing solely on one-time exam performance, may encourage students to shift their goals toward deeper learning. Given that fully changing students’ mindsets away from grade-oriented goals may be difficult, it could also be beneficial to frame effective strategies in terms of both learning benefits and potential grade improvements. For example, future interventions could make more explicit how using these strategies may lead to better performance on exams—an outcome many students value. Future research should examine alternative ways of creating curricula that prioritizes long-term mastery while still appealing to students’ performance-related motivations.

Another valuable finding from the open-ended questions was that even among students who were more aligned with the intervention’s goal of long-term retention, many struggled with implementing effective strategies in their daily study routines. A common challenge they faced was feeling limited in their available time. Students frequently mentioned that time constraints made it difficult to engage in more effortful but effective strategies, such as spaced learning. To address these challenges, some students mentioned using time management tools; however, others struggled with the additional effort required to plan their studying. Explicit instruction in time management could be a key component of future interventions aimed at promoting strategies like spacing and interleaving.

While addressing these barriers within the intervention is one way to produce further behavioral changes, some obstacles may also require fostering a broader learning environment that actively supports good practices across multiple classrooms. In particular, the low reported frequency of use of some learning strategies may not reflect a lack of understanding or motivation, but rather a lack of access to the necessary resources. For instance, because limited access—especially for retrieval practice—was frequently cited as a barrier, instructors could incorporate cumulative weekly quizzes into coursework to provide structured self-testing opportunities while also facilitating interleaving and spacing. Addressing these resource limitations outside the intervention itself may therefore be key to increasing the use of effective strategies. When students consistently engage with these practices in the context of their regular coursework, they are more likely to develop long-lasting habits that support sustained learning (see for review Zimmerman, 2002). In turn, the impact of interventions like ours may be further strengthened when reinforced across different learning environments.

Despite the effectiveness of the intervention and the valuable insights gained from this study, it is also important to acknowledge its limitations. Although we reached out to a large pool of students, the final sample size was small relative to the entire eligible population as well as relative to the previously published work. Moreover, while the participation rates for both the intervention and business as usual groups were within a few percentage points of each other, because the rate was slightly higher in the intervention group, additional caution with regard to drawing strong causal inferences regarding the impact of the intervention is warranted. The voluntary nature of participation may also have introduced selection bias, as those who enrolled could differ in meaningful ways from the broader student population. Although the data suggest that there is strong commonality between at least the subset of participants who participated in the current work and in the Yüksel et al. (2024) study, these factors nonetheless limit the generalizability of our findings. Additionally, while the natural setting of this study allowed us to assess the intervention’s real-world impact across five years, it also meant that we could not track participants as systematically as in a controlled experiment. Furthermore, given the relatively small sample size, we were cautious not to overinterpret differences at the level of individual learning strategies. Given the many nuances associated with individual strategies (e.g., where the degree of effectiveness may depend on implementation or contextual details), future research should prioritize methods that allow for deeper strategy-level analyses.

Another limitation of the current design is the potential for order effects, as the perceived effectiveness ratings were collected prior to reported frequency of use. This raises the possibility that participants’ judgments of effectiveness may have influenced how frequently they reported using each strategy. While we did observe a number of cases where ratings of effectiveness and frequency of use diverged (e.g., cramming was rated as relatively ineffective, but also commonly used), it is possible that these are underestimates of the true degree of divergence. Because the sheer number of items makes full counterbalancing impossible, it would be valuable for future work to consider alternative approaches to reduce the possibility that order effects play a role in observed results (e.g., by counterbalancing effectiveness ratings and frequency of use ratings, without item-level counterbalancing). Finally, we measured only the self-reported frequency of strategy use; future studies should aim to capture actual behavioral changes. Future research might benefit from longitudinal designs that follow individual students over time to better capture changes in understanding, strategy use, and obstacles.

In conclusion, our findings demonstrate that while it is possible to foster sustainable changes in students’ understanding and use of effective learning strategies, achieving long-term behavioral change requires addressing both cognitive and logistical barriers. Future interventions should educate students on effective strategies and provide concrete tools, support, and reinforcement to help them integrate these strategies into their routines. Furthermore, instructors play a crucial role in embedding these strategies into coursework in order to make their adoption easier and more enduring. Actively engaging students in the learning process and reinforcing these strategies over time can support their consistent use. By shifting the focus from short-term performance to long-term mastery, future efforts can bridge the gap between knowledge and behavior, ultimately fostering better self-regulated learning practices.

## Figures and Tables

**Table 1:** Mean (standard deviation) of perceived effectiveness ratings for effective and ineffective learning strategies across groups and time points in Study 1.

Perceived Effectiveness						
	Effective Strategies			Ineffective Strategies		
Groups	Pre- Intervention	Post- Intervention	Long-Term Follow-Up	Pre- Intervention	Post- Intervention	Long-Term Follow-Up
Business-as-usual	7.83 (1.44)	7.59 (1.45)	7.14 (1.39)	6.80 (1.42)	5.97 (1.41)	5.96 (1.32)
Intervention	7.24 (1.92)	8.05 (1.36)	7.87 (1.25)	6.31 (1.63)	4.28 (1.44)	4.49 (1.16)

**Table 2:** Mean (standard deviation) of reported frequency of use ratings for effective and ineffective learning strategies across groups and time points in Study 1.

Reported Frequency of Use						
	Effective Strategies			Ineffective Strategies		
Groups	Pre- Intervention	Post- Intervention	Long-Term Follow-Up	Pre- Intervention	Post- Intervention	Long-Term Follow-Up
Business-as-usual	6.68 (1.27)	5.33 (1.76)	5.39 (1.48)	6.40 (1.37)	5.74 (1.55)	5.78 (1.21)
Intervention	6.55 (1.97)	5.73 (1.36)	6.34 (1.13)	6.70 (1.73)	4.74 (1.29)	5.39 (1.35)

**Table 3:** Mean (standard deviation) of perceived effectiveness ratings for effective and ineffective learning strategies across groups and time points in Study 2.

	Perceived Effectiveness		Reported Frequency of Use		Scenarios
Groups	Effective Strategies	Ineffective Strategies	Effective Strategies	Ineffective Strategies	
Business-as-usual	6.79 (1.22)	5.48 (1.47)	5.62 (1.38)	5.60 (1.34)	6.80 (1.02)
Intervention	7.83 (0.96)	4.50 (1.63)	6.51 (1.29)	5.04 (1.52)	8.28 (1.18)

**Table 4:** Frequency and percentage of reported learning strategies by group.

Response	Business-as-Usual	Intervention	Total
Retrieval Practice	37/76 (49%)	15/35 (43%)	52/111 (47%)
Rereading	33/76 (43%)	14/35 (40%)	47/111 (42%)
Cramming	21/76 (28%)	8/35 (23%)	29/111 (26%)
Time Management	22/76 (29%)	7/35 (20%)	29/111 (26%)
Spacing	20/76 (26%)	8/35 (23%)	28/111 (25%)
Using Flashcards	22/76 (29%)	4/35 (11%)	26/111 (23%)
Studying in Advance	6/76 (8%)	9/35 (26%)	15/111 (14%)
Copying Notes	8/76 (11%)	4/35 (11%)	12/111 (11%)
Creating Outline	8/76 (11%)	0/35 (0)	8/111 (7%)
Group Study	4/76 (5%)	3/35 (9%)	7/111 (6%)
Active Notetaking	2/76 (3%)	4/35 (11%)	6/111 (5%)
Metacognitive Monitoring	3/76 (4%)	3/35 (9%)	6/111 (5%)
Watching Videos	4/76 (5%)	2/35 (6%)	6/111 (5%)
Self-Explanation	3/76 (4%)	1/35 (3%)	4/111 (4%)
Summarization	3/76 (4%)	1/35 (3%)	4/111 (4%)
Using Planner	2/76 (3%)	1/35 (3%)	3/111 (3%)
Visualization	2/76 (3%)	1/35 (3%)	3/111 (3%)
Environment Optimization	1/76 (1%)	1/35 (3%)	2/111 (2%)
Error Analysis	2/76 (3%)	0/35 (0)	2/111 (2%)
Attending Office Hours	1/76 (1%)	1/35 (3%)	2/111 (2%)
Studying in Different Places	0/76 (0)	2/35 (6%)	2/111 (2%)
Highlighting	1/76 (1%)	0/35 (0)	1/111 (1%)
Learning From Examples	1/76 (1%)	0/35 (0)	1/111 (1%)
Listening Audiobooks	1/76 (1%)	0/35 (0)	1/111 (1%)
Interleaved Practice	0/76 (0)	1/35 (3%)	1/111 (1%)

**Table 5:** Frequency and percentage of students’ perceptions of the effectiveness of their own learning strategies: are they effective? (yes, unsure, no).

Response	Business-as-Usual	Intervention	Total
Yes	58/80 (73%)	28/37 (76%)	86/117 (74%)
Unsure	15/80 (19%)	4/37 (11%)	19/117 (16%)
No	7/80 (9%)	5/37 (14%)	12/117 (10%)

**Table 6:** Frequency and percentage of reported reasons for perceived strategy ineffectiveness.

Response	Business-as-Usual	Intervention	Total
There Are More Effective Strategies	11/17 (64.7%)	2/6 (33.3%)	13/23 (56.5%)
Procrastination	3/17 (17.6%)	3/6 (50.0%)	6/23 (26.1%)
Not Organized	4/17 (23.5%)	1/6 (16.7%)	5/23 (21.7%)
Lack of Information	3/17 (17.6%)	0/6 (0.0%)	3/23 (13.0%)

**Table 7:** Frequency and percentage of reported reasons for perceived strategy effectiveness.

Response	Business-as-Usual	Intervention	Total
Good Grades	22/65 (33.8%)	10/32 (31.3%)	32/97 (33.0%)
Learn Effectively	17/65 (26.2%)	14/32 (43.8%)	31/97 (32.0%)
Understand, Not Memorize	12/65 (18.5%)	4/32 (12.5%)	16/97 (16.5%)
Good Representation of Exams	7/65 (10.8%)	2/32 (6.3%)	9/97 (9.3%)
Helps with Thinking Through Problems	4/65 (6.2%)	4/32 (12.5%)	8/97 (8.2%)
Save Time	5/65 (7.7%)	3/32 (9.4%)	8/97 (8.2%)
Feels Productive	5/65 (7.7%)	2/32 (6.3%)	7/97 (7.2%)
Stay Organized	5/65 (7.7%)	0/32 (0.0%)	5/97 (5.2%)
Help Fix Weaknesses	0/65 (0.0%)	2/32 (6.3%)	2/97 (2.1%)

**Table 8:** Frequency and percentage of reported intended changes to learning strategies.

Response	Business-as-Usual	Intervention	Total
Retrieval Practice	30/76 (39.5%)	17/36 (47.2%)	47/112 (42.0%)
Time Management	23/76 (30.3%)	11/36 (30.6%)	34/112 (30.4%)
Using Flashcards	25/76 (32.9%)	7/36 (19.4%)	32/112 (28.6%)
Rereading	20/76 (26.3%)	10/36 (27.8%)	30/112 (26.8%)
Spacing	12/76 (15.8%)	8/36 (22.2%)	20/112 (17.9%)
Group Study	13/76 (17.1%)	5/36 (13.9%)	18/112 (16.1%)
Copying Notes	13/76 (17.1%)	4/36 (11.1%)	17/112 (15.2%)
Study More	10/76 (13.2%)	4/36 (11.1%)	14/112 (12.5%)
Studying Different Places	5/76 (6.6%)	6/36 (16.7%)	11/112 (9.8%)
Studying in Advance	8/76 (10.5%)	3/36 (8.3%)	11/112 (9.8%)
Environment Optimization	6/76 (7.9%)	4/36 (11.1%)	10/112 (8.9%)
Creating Outline	7/76 (9.2%)	2/36 (5.6%)	9/112 (8.0%)
Metacognitive Monitoring	7/76 (9.2%)	2/36 (5.6%)	9/112 (8.0%)
Visualization	6/76 (7.9%)	3/36 (8.3%)	9/112 (8.0%)
Self-Explanation	3/76 (3.9%)	5/36 (13.9%)	8/112 (7.1%)
Active Note-Taking	2/76 (2.6%)	5/36 (13.9%)	7/112 (6.3%)
Office Hours	7/76 (9.2%)	0/36 (0.0%)	7/112 (6.3%)
Summarization	6/76 (7.9%)	1/36 (2.8%)	7/112 (6.3%)
Using Planner	6/76 (7.9%)	1/36 (2.8%)	7/112 (6.3%)
Watching Videos	5/76 (6.6%)	1/36 (2.8%)	6/112 (5.4%)
Cramming	4/76 (5.3%)	0/36 (0.0%)	4/112 (3.6%)
Interleaved Practice	1/76 (1.3%)	3/36 (8.3%)	4/112 (3.6%)
Highlighting	3/76 (3.9%)	0/36 (0.0%)	3/112 (2.7%)
Real-Life Examples	3/76 (3.9%)	0/36 (0.0%)	3/112 (2.7%)
Mnemonics	1/76 (1.3%)	1/36 (2.8%)	2/112 (1.8%)
Listening to Audiobooks	1/76 (1.3%)	0/36 (0.0%)	1/112 (0.9%)
Elaborative Interrogation	0/76 (0.0%)	1/36 (2.8%)	1/112 (0.9%)
Error Analysis	0/76 (0.0%)	1/36 (2.8%)	1/112 (0.9%)
Learning from Examples	0/76 (0.0%)	1/36 (2.8%)	1/112 (0.9%)

**Table 9:** Frequency and percentage of reported obstacles to implementing learning strategies.

Response	Business-as-Usual	Intervention	Total
Not Enough Time	31/67 (46.3%)	17/30 (56.7%)	48/97 (49.5%)
No Desire	21/67 (31.3%)	9/30 (30.0%)	30/97 (30.9%)
Lack of Resources	14/67 (20.9%)	5/30 (16.7%)	19/97 (19.6%)
Unsure Effectiveness	9/67 (13.4%)	5/30 (16.7%)	14/97 (14.4%)
Procrastination	9/67 (13.4%)	3/30 (10.0%)	12/97 (12.4%)
Distractions	7/67 (10.4%)	4/30 (13.3%)	11/97 (11.3%)
Hard to Change	4/67 (6.0%)	5/30 (16.7%)	9/97 (9.3%)
Nobody Else Available	2/67 (3.0%)	4/30 (13.3%)	6/97 (6.2%)
Mental Health	5/67 (7.5%)	0/30 (0.0%)	5/97 (5.2%)
Schedule Conflicts	2/67 (3.0%)	3/30 (10.0%)	5/97 (5.2%)
Lack of Flow	3/67 (4.5%)	1/30 (3.3%)	4/97 (4.1%)
Variety Requirement	2/67 (3.0%)	2/30 (6.7%)	4/97 (4.1%)
Distance	3/67 (4.5%)	0/30 (0.0%)	3/97 (3.1%)
Introversion	3/67 (4.5%)	0/30 (0.0%)	3/97 (3.1%)
Monetary Restrictions	1/67 (1.5%)	1/30 (3.3%)	2/97 (2.1%)

## Data Availability

The original data presented in the study and the scripts for data analyses are openly available in https://osf.io/kcduz/?view_only=8ba58424b68743c9a39a6e7b66c34516 accessed on 24 April 2025.

## Appendix A

### Appendix A.1

**Table A1:** Perceived effectiveness ratings for each learning strategy in Study 1.

Learning Strategies	Intervention			Business-as-Usual			Group × Time *p*-Value
	Pre- Intervention	Post- Intervention	Long-Term	Pre- Intervention	Post- Intervention	Long-Term	
Reading Textbook	6.36	4.06	2.88	6.79	6.69	5.83	0.052
Taking Tests	8.36	9.88	9.71	9.15	9.53	9.28	0.046 *
Using Life Examples	7.82	9.12	8.29	8.06	8.62	7.91	0.547
Looking at Notes	7.91	6.59	5.29	8.41	7.49	7.91	0.030 *
Highlighting Notes	5.64	2.53	3.29	7.15	4.51	5.19	0.773
Creating Outlines	6.27	6.41	6.53	7.76	5.38	7.02	0.588
Copying Notes	6.00	4.94	5.53	6.53	6.09	6.91	0.526
Highlighting Textbook	5.73	1.71	2.12	6.47	4.20	4.63	0.150
Cramming	4.73	3.29	3.65	3.79	3.84	3.85	0.421
Summarizing Notes	8.00	7.00	8.35	8.38	7.76	7.85	0.283
Using Flashcards	6.82	7.12	7.24	7.59	7.76	7.59	0.718
Group Study	6.36	7.65	6.88	6.76	6.11	5.98	0.365

Each strategy was analyzed using a linear mixed-effects model with time (coded as pre-intervention, post-intervention, and long-term follow-up), section (intervention and business-as-usual) and the interaction term as fixed effects, and a random intercept for part. An asterisk (*) indicates that the *p*-value is statistically significant.

**Table A2:** Reported frequency of use for each learning strategy in Study 1.

Learning Strategies	Intervention			Business-as-Usual			Group × Time *p*-Value
	Pre- Intervention	Post- Intervention	Long-Term	Pre- Intervention	Post- Intervention	Long-Term	
Reading Textbook	5.91	3.59	3.41	6.24	6.24	4.74	0.59
Taking Tests	8.00	9.52	8.47	8.50	7.53	7.21	0.17
Using Life Examples	6.82	7.76	7.12	7.79	6.75	6.15	0.09
Looking at Notes	8.91	8.00	8.35	8.47	8.46	8.33	0.74
Highlighting Notes	6.00	2.47	4.00	7.15	4.77	4.37	0.47
Creating Outlines	5.90	4.00	5.35	6.64	4.48	5.04	0.42
Copying Notes	5.45	5.53	6.41	5.09	4.93	6.35	0.81
Highlighting Textbook	5.81	1.23	2.11	5.14	2.31	2.54	0.44
Cramming	6.00	6.35	6.17	5.44	7.72	6.84	0.36
Summarizing Notes	7.36	5.94	7.35	7.70	6.71	6.35	0.19
Using Flashcards	5.54	3.64	4.47	5.50	5.68	4.73	0.99
Group Study	5.82	3.53	5.29	5.18	3.93	4.13	0.57

For both the business-as-usual and intervention groups, assessment time points are labeled based on their timing relative to the intervention period. “Pre-intervention” refers to assessments conducted before the intervention occurred, “post-intervention” to those conducted immediately after, and “long-term” to those conducted approximately six months later. Each strategy was analyzed using a linear mixed-effects model with time (coded as pre-intervention, post-intervention, and long-term follow-up), section (intervention and business-as-usual) and the interaction term as fixed effects, and a random intercept for time.

### Appendix A.2

**Table A3:** Responses to open-ended questions: obstacles in Study 1.

Response	Business-as-Usual	Intervention	Total
Not Enough Time	2	0	2
No Desire	1	0	1
Lack of Resources	4	1	5
Unsure Effectiveness	13	3	16
Procrastination	3	1	4
Distractions	11	5	16
Hard to Change	2	0	2
Nobody Else Available	1	1	2
Mental Health	1	2	3

**Table A4:** Responses to open-ended questions: current strategies in Study 1.

Response	Business-as-Usual	Intervention	Total
Retrieval Practice	8	3	11
Rereading	1	1	2
Cramming	5	0	5
Time Management	3	1	4
Spacing	1	0	1
Using Flashcards	3	1	4
Studying in Advance	9	3	12
Copying Notes	5	0	5
Creating Outline	2	1	3
Group Study	1	0	1
Active Notetaking	5	0	5
Metacognitive Monitoring	1	0	1
Watching Videos	3	0	3
Self-Explanation	13	5	18
Summarization	3	1	4
Using Planner	4	2	6
Visualization	3	0	3
Environment Optimization	1	0	1
Error Analysis	1	0	1
Attending Office Hours	5	0	5
Studying in Different Places	3	1	4
Highlighting	1	0	1
Learning From Examples	1	0	1
Listening Audiobooks	1	0	1
Interleaved Practice	1	0	1

## Appendix B

### Appendix B.1

**Table A5:** Number of participants per semester, broken down by group.

	Number of Participants		
Semester	Business-as-Usual	Intervention	Total
2019–2020 Fall	10	0	10
2019–2020 Spring	2	0	2
2020–2021 Fall	10	5	15
2020–2021 Spring	2	3	5
2021 Summer	2	0	2
2021–2022 Fall	15	4	19
2021–2022 Spring	9	0	9
2022 Summer	1	0	1
2022–2023 Fall	8	22	30
2022–2023 Spring	9	0	9

### Appendix B.2

**Table A6:** Perceived effectiveness ratings for each learning strategy in Study 2.

Learning Strategies	Intervention	Business-as-Usual	*p* Value
Reading Textbook	3.74	5	0.87
Looking at Notes	6.41	7.44	0.94
Copying Notes	5.24	6.1	0.20
Summarizing Notes	6.44	7.12	0.80
Taking Tests	9.26	8.81	0.06
Highlighting Notes	3.09	4.53	0.23
Highlighting Textbook	2.79	3.66	0.73
Using Flashcards	7.09	6.87	0.24
Using Life Examples	8.29	7.5	0.10
Creating Outlines	7	6.53	0.098
Cramming	3.82	4.54	0.278
Group Study	7.32	5.6	0.01 *
Studying in Different Places	8.15	5.81	0.0001 **
Spacing	8.15	7.29	0.45
Self-Explanation	9.29	8.59	0.13
Interleaving	5.88	4.37	0.002 **

A double asterisk (**) indicates statistical significance at *p* < 0.001, and a single asterisk (*) indicates statistical significance at *p* < 0.05.

**Table A7:** Reported frequency of use for each learning strategy in Study 2.

Learning Strategies	Intervention	Business-as-Usual	*p* Value
Reading Textbook	4.24	4.43	0.17
Looking at Notes	8.15	8.35	0.86
Copying Notes	5.59	5.82	0.48
Summarizing Notes	5.47	5.99	0.69
Taking Tests	8.44	7.46	0.15
Highlighting Notes	3.06	5.04	0.21
Highlighting Textbook	2.5	3.18	0.71
Using Flashcards	5.12	5.29	0.76
Using Life Examples	6.62	6.32	0.73
Creating Outlines	5.56	5.49	0.57
Cramming	6.29	6.43	0.51
Group Study	5.62	4.35	0.11
Studying in Different Places	7.29	5.37	0.01 *
Spacing	6.44	5.84	0.56
Self-Explanation	6.88	5.79	0.14
Interleaving	6.41	4.79	0.007 **

A double asterisk (**) indicates statistical significance at *p* < 0.001, and a single asterisk (*) indicates statistical significance at *p* < 0.05.
